# Characterization of Pik1 function in fission yeast reveals its conserved role in lipid synthesis and not cytokinesis

**DOI:** 10.1242/jcs.261415

**Published:** 2023-11-01

**Authors:** Alaina H. Willet, Lesley A. Turner, Joshua S. Park, Liping Ren, Chloe E. Snider, Kathleen L. Gould

**Affiliations:** Department of Cell and Developmental Biology, Vanderbilt University School of Medicine, Nashville, TN 37232, USA

**Keywords:** PI4P, PI(4,5)P_2_, Lipid kinase, Pik1, Fission yeast, Cytokinesis

## Abstract

Phosphatidylinositol (PI)-4-phosphate (PI4P) is a lipid found at the plasma membrane (PM) and Golgi in cells from yeast to humans. PI4P is generated from PI by PI4-kinases and can be converted into PI-4,5-bisphosphate [PI(4,5)P_2_]. *Schizosaccharomyces pombe* have two essential PI4-kinases – Stt4 and Pik1. Stt4 localizes to the PM, and its loss from the PM results in a decrease of PM PI4P and PI(4,5)P_2_. As a result, cells divide non-medially due to disrupted cytokinetic ring–PM anchoring. However, the localization and function of *S. pombe* Pik1 has not been thoroughly examined. Here, we found that Pik1 localizes exclusively to the trans-Golgi and is required for Golgi PI4P production. We determined that Ncs1 regulates Pik1, but unlike in other organisms, it is not required for Pik1 Golgi localization. When Pik1 function was disrupted, PM PI4P but not PI(4,5)P_2_ levels were reduced, a major difference compared with Stt4. We conclude that Stt4 is the chief enzyme responsible for producing the PI4P that generates PI(4,5)P_2_. Also, that cells with disrupted Pik1 do not divide asymmetrically highlights the specific importance of PM PI(4,5)P_2_ for cytokinetic ring–PM anchoring.

## Teaser

**Summary:** Fission yeast Pik1 localizes exclusively to the trans-Golgi independently of Ncs1, where it contributes to PI4P but not PI(4,5)P_2_ synthesis. Pik1 does not affect cytokinesis.

## INTRODUCTION

Phosphoinositides (PIPs) are abundant lipid species that are important for a variety of cellular processes including cell division and membrane trafficking (Echard, 2012; Schuh and Audhya, 2012). Phosphatidylinositol (PI)-4-phosphate (PI4P), which is made by PI-4-kinases phosphorylating the head group of PI, is a precursor for PI-4,5-bisphosphate [PI(4,5)P_2_] and both lipid species are important for cell division in diverse organisms (Emoto et al., 2005; Field et al., 2005; Snider et al., 2017, 2018).

**Fig. 1.**
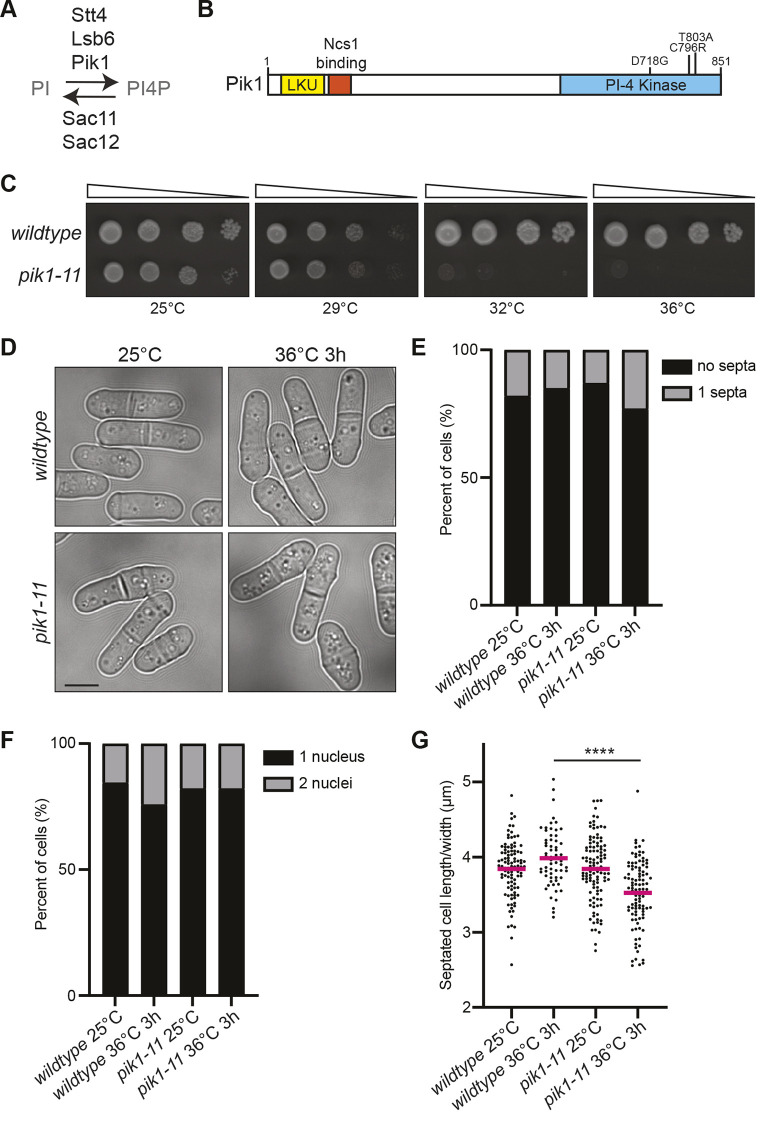
**Isolation and characterization of *pik1-11*.** (A) A schematic of *S. pombe* proteins known to phosphorylate PI to convert it into PI4P or to dephosphorylate PI4P. (B) A schematic of Pik1, drawn to scale, with the domains and mutations encoded by *pik1-11* labelled. LKU, lipid kinase unique; PI-4 kinase, phosphatidylinositol-4-kinase. (C) 10-fold serial dilutions of the indicated strains grown at the indicated temperatures for 3–4 days on YE agar. (D) Differential interference contrast (DIC) live-cell images of wild-type and *pik1-11* cells grown at 25°C and shifted to 36°C for 3 h. Images were acquired at both time points. Scale bar: 5 μm. (E) Quantification of the septation index of the indicated strains at the indicated temperatures. *n*>400 for each from two biological replicates. (F) Cells were grown up as in D and fixed and stained with DAPI and methyl blue (MB). Nuclei/cell were quantified. *n*≥270 for each from two biological replicates. (G) Cells grown as in D were measured for the ratio of septated cell length divided by the cell width. *n*≥62 for each from two biological replicates. Line represents mean. ****P≤0.0001 (one-way ANOVA with Tukey's post-test). Images in C and D are representative of two repeats.

The fission yeast, *Schizosaccharomyces pombe*, has three PI-4-kinases: Stt4, Lsb6 and Pik1, and two PI4 phosphatases, Sac11 and Sac12 (Fig. 1A). Stt4 is an essential type IIIα enzyme that localizes to the PM via two scaffolds, Efr3 and Ypp1 (Baird et al., 2008; Snider et al., 2017). Efr3 is critical for Stt4 PM localization and *efr3Δ* cells have reduced PM PI4P and PI(4,5)P_2_ (Snider et al., 2017). For *S. pombe* cells to divide, they build an actin- and myosin-based cytokinetic ring (CR) at the cell middle that guides the deposition of a medial septum (Marks et al., 1986; Kitayama et al., 1997). Normally the CR remains anchored at its original medial position so that upon CR constriction and septation, two daughter cells of equal size are produced (Snider et al., 2017). However, *efr3Δ* cells divide asymmetrically because the CR is not properly anchored and it slides toward one cell end prior to constriction and septation (Snider et al., 2017). It has been hypothesized that PI(4,5)P_2_ is particularly important for CR anchoring; however, a role for PI4P in this process could not be ruled out (Snider et al., 2017, 2018).

Lsb6 is a type II PI-4-kinase that localizes to the vacuolar membrane (Matsuyama et al., 2006). Cells lacking *lsb6* do not have any reported cellular defects (Kim et al., 2010), however, they do have a reduction in PM PI4P and a negative genetic interaction with *efr3Δ* cells (Snider et al., 2018).

Finally, Pik1 is an essential type IIIβ PI-4-kinase that has been extensively studied in *Saccharomyces cerevisiae*. In *S. cerevisiae*, Pik1 localizes to the Golgi, where it generates PI4P (Flanagan et al., 1993; Walch-Solimena and Novick, 1999) and is essential for proper Golgi to PM protein trafficking (Hama et al., 1999; Walch-Solimena and Novick, 1999). Pik1 complexes with the Ca^2+^-binding protein Frq1, which is important for Pik1 kinase activity and Golgi localization (Hendricks et al., 1999; Ames et al., 2000; Strahl et al., 2005; Lim et al., 2011). Less is known about *S. pombe* Pik1. It is reported to localize to the Golgi, but also the cell division site, a localization not reported for Pik1 orthologs in other organisms (Park et al., 2009). *S. pombe* Pik1 has also been reported to bind the Frq1 ortholog, Ncs1, which is thought to regulate its activity (Hamasaki-Katagiri et al., 2004). Interestingly, *S. pombe* Pik1 has also been reported to be involved in cytokinesis and to bind the myosin light chains Cam2 and Cdc4 via a C-terminal pseudo isoleucine-glutamine (IQ) motif (Desautels et al., 2001; Sammons et al., 2011). These interactions are not reported for Pik1 orthologs in other species, and the function of these potential interactions has been unclear.

Here, we aimed to clarify Pik1 function in *S. pombe* by constructing and characterizing a *pik1* temperature-sensitive allele, which we named *pik1-11*, and an endogenous fluorescently tagged *pik1* allele. Our results indicate that *S. pombe* Pik1 functions as the sole Golgi-localized PI4-kinase. Furthermore, it does not contribute substantially to the PI4P precursors that are converted into PI(4,5)P_2_. Importantly, we found no evidence that *S. pombe* Pik1 colocalizes with Cdc4 or Cam2 or contributes to cytokinesis, but Ncs1 is indeed an important regulator of Pik1.

## RESULTS AND DISCUSSION

To investigate the function of Pik1, an essential PI-4-kinase, we constructed a temperature-sensitive allele, which we named *pik1-11* (Fig. 1B,C). The protein encoded by *pik1-11* contains three point mutations within the PI-4-kinase domain (Fig. 1B). *pik1-11* cells grow similarly to wild-type cells at 25°C and 29°C but *pik1-11* cells do not grow at 32°C or 36°C (Fig. 1C). A previously described *pik1* allele, *pik1-td*, displayed defects in cell division (Park et al., 2009). In contrast, we did not observe any difference in the septation index of *pik1-11* cells at permissive or restrictive temperatures compared to wild type (Fig. 1D,E). We also did not observe an accumulation of multi-nucleated cells (Fig. 1F). Thus, we did not observe any cellular defect indicating a role for Pik1 in cell division. However, the morphology of *pik1-11* cells changed at the non-permissive temperature; they became shorter and wider (Fig. 1G).

**Fig. 2.**
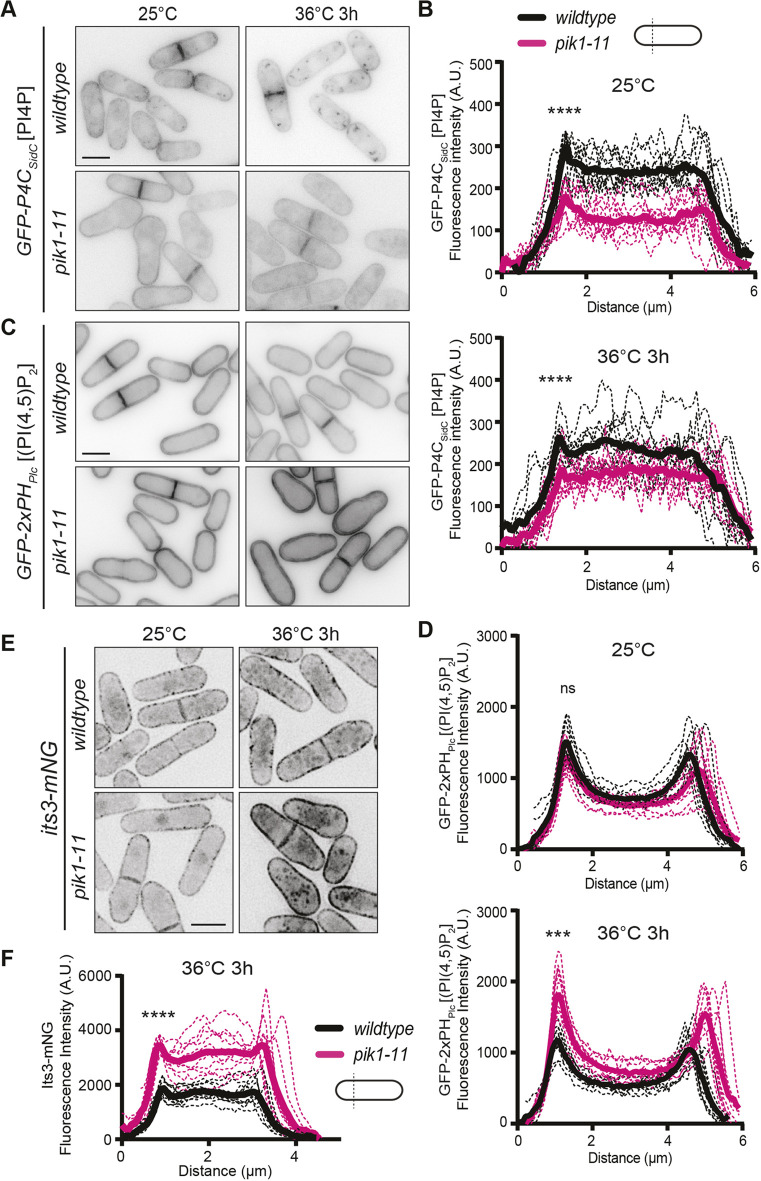
**Analysis of lipid levels and septa position in *pik1-11*.** Live-cell images of GFP–P4C_SidC_ (A) and GFP–2xPH_Plc_ (C) in wild-type and *pik1-11* cells. Cells were grown at 25°C and shifted to 36°C for 3 h. Images were acquired at both time points. (B,D,F) Line scans of fluorescence intensity drawn across the short axis of 10 cells for each indicated strain at the indicated temperature. Solid lines represent the mean and dotted lines are the individual line traces. Data is from two biological replicates. For B, wild-type versus *pik1-11* cells at 25°C at first peak, 1.51 μm distance, is *P*<0.0001, and wild-type versus *pik1-11* cells at 36°C at first peak, 1.29 μm distance, is *P*=0.0001. For D, wild-type versus *pik1-11* cells at 25°C at first peak, 1.29 μm distance, is *P*=0.055 and wild-type versus *pik1-11* cells at 36°C at first peak, 1.08 μm distance, is *P*=0.0003. For F, wild-type versus *pik1-11* cells at first peak, 0.93 μm distance, is *P*<0.0001. ****P≤0.0001, ***P≤0.001; ns, not significant (unpaired two-tailed Student's *t*-test). (E) Live-cell images of Its3–mNG in wild-type and *pik1-11* cells. Cells were grown at 25°C and shifted to 36°C for 3 h and imaged at both time points. Images in E are representative of two repeats. A.U., arbitrary units. Scale bars: 5 μm.

Because Pik1 converts PI into PI4P, we investigated cellular PI4P levels with the established GFP-tagged lipid biosensor GFP–P4C_SidC_ (Luo et al., 2015; Snider et al., 2017). In wild-type cells at 25°C and after 3 h at 36°C, GFP–P4C_SidC_ localized to the PM and internal puncta that correspond to the Golgi (Fig. 2A). GFP–P4C_SidC_ in *pik1-11* cells did not show any detectable Golgi localization, and the PM intensity was reduced compared to wild-type cells at both permissive and restrictive temperatures (Fig. 2A,B). Because PI4P is a precursor of PI(4,5)P_2_, we next analyzed cellular PI(4,5)P_2_ localization with the GFP–2xPH_Plc_ lipid biosensor (Stefan et al., 2002; Snider et al., 2017). At 25°C, GFP–2xPH_Plc_ showed the expected PM localization in wild-type and in *pik1-11* cells there was no difference in cortical fluorescence intensity compared to wild type (Fig. 2C,D). At 36°C, there was an ∼65% increase in cortical GFP–2xPH_Plc_ localization in *pik1-11* compared to wild-type cells (Fig. 2C,D). We conclude that *pik1-11* cells lack a Golgi PI4P pool, and have reduced PM levels of PI4P, but that does not result in a corresponding decrease in PM PI(4,5)P_2_.

That *pik1-11* cells had reduced PM PI4P but not reduced PI(4,5)P_2_ was an intriguing observation. Other existing gene deletions and temperature-sensitive alleles involved in PI4P synthesis (*efr3Δ*, *its3-1*) have reduced PM PI4P and also reduced PM PI(4,5)P_2_ (Snider et al., 2017, 2018), thus making dissecting the contributions of each lipid challenging. It has been previously hypothesized that the CR anchoring defects of *efr3Δ* cells was most likely due to reduced PI(4,5)P_2_ rather than a change in PI4P because many CR proteins specifically bind PI(4,5)P_2_ (Cauvin and Echard, 2015; McDonald et al., 2015; Sun et al., 2015). The *pik1-11* allele allowed us to test this hypothesis more directly. We measured the septa position of wild-type and *pik1-11* cells at the permissive and restrictive temperatures and did not observe a difference in *pik1-11* compared to wild type in any condition (Fig. S1A). This result is consistent with the hypothesis that altering PI4P levels alone do not impact CR–PM anchoring. Similarly, *lsb6Δ* cells have reduced PM PI4P levels and do not have off-centered septa (Snider et al., 2018). However, it was not reported whether *lsb6Δ* cells have any changes in PM PI(4,5)P_2_ levels. To test this, we compared wild-type and *lsb6Δ* cells expressing GFP–2xPH_Plc_ and found no difference in the cortical fluorescence intensity of the PI(4,5)P_2_ sensor in these strains (Fig. S1B,C). Overall, these results suggest that Stt4, but not Pik1 or Lsb6, generates the PI4P at the PM that is subsequently converted into PI(4,5)P_2._ This conclusion is in accord with the exclusive localization of the PI4-5-kinase, Its3, to the PM (Zhang et al., 2000).

We next wanted to better understand why *pik1-11* cells have increased PM PI(4,5)P_2_ at the restrictive temperature. Given that disruption of *pik1* causes protein trafficking defects in *S. cerevisiae* (Hama et al., 1999; Walch-Solimena and Novick, 1999), we reasoned that *S. pombe* proteins involved in PI(4,5)P_2_ synthesis could be mislocalized. Specifically, an increase in PM Its3 or Stt4 and/or a decrease in PM PI4-5-phosphatases could account for the observed increased in PM PI(4,5)P_2_. We found no change in the localization of GFP–Stt4, or the PI4-5-phosphatases Inp53-mNG and Syj1-mNG (Snider et al., 2018) in *pik1-11* cells compared to wild-type cells at 25°C or 36°C (Fig. S1D). In contrast, PM levels of Its3–mNG were mildly reduced at 25°C and increased 1.8-fold at 36°C in *pik1-11* compared to wild-type cells (Fig. 2E,F; Fig. S1E). We conclude that the increased PM PI(4,5)P_2_ and decreased PM PI4P observed in *pik1-11* cells at the restrictive temperature can be explained by additional Its3 activity at the PM.

We also tested whether there were genetic interactions between *pik1-11* and other components of the PIP pathway. No genetic interaction was observed with *GFP-stt4*, a hypomorphic *stt4* allele (Snider et al., 2017) or *lsb6Δ* (Fig. S2A). We did find a negative genetic interaction with *efr3Δ* and *its3-1*, but not with a deletion of *sac12*, a gene encoding a PI-4-phosphatase (Harris et al., 2022) (Fig. S2A). Overall, we conclude that the PI4-kinases in *S. pombe* function independently, as in *S. cerevisiae* (Strahl et al., 2005), and disruption of other genes important for PI(4,5)P_2_ generation leads to further growth defects of *pik1-11* cells.

**Fig. 3.**
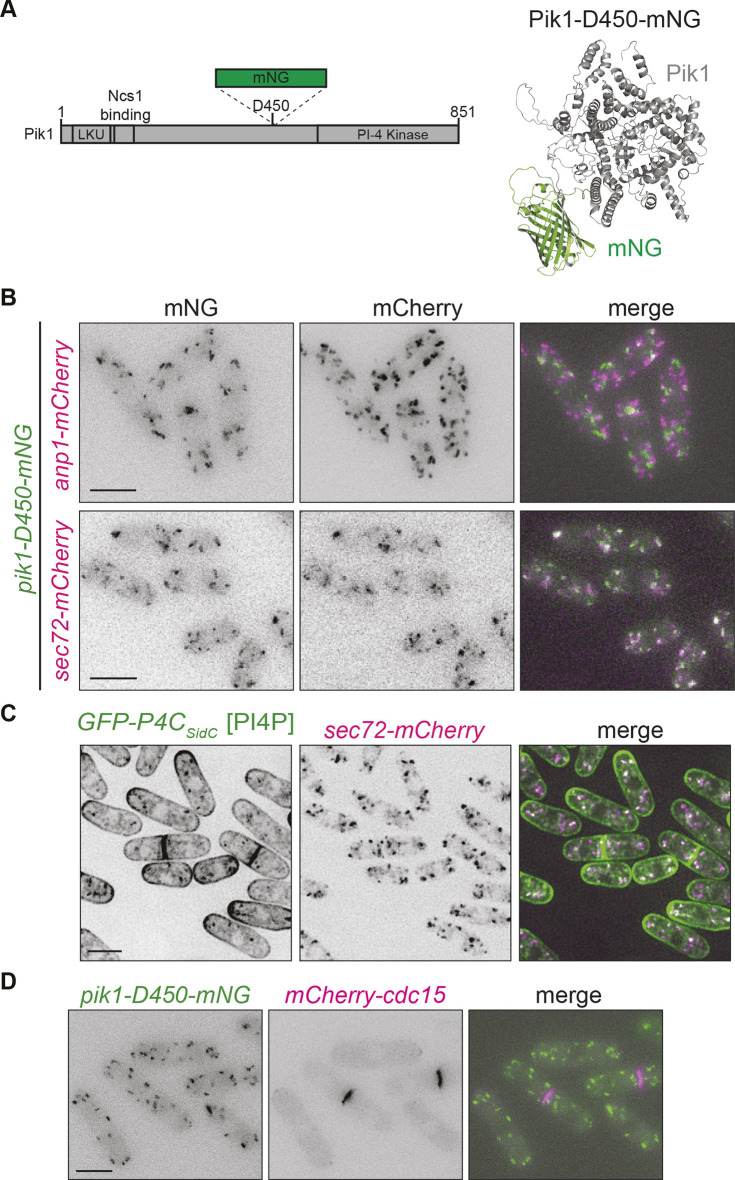
**Pik1 localizes exclusively to the trans-Golgi.** (A) Left, a schematic of Pik1 drawn to scale with domains, mNG and the insertion site labelled. Right, AlphaFold2 predicted structure of Pik1 with mNG inserted after residue D450. Pik1 is gray and mNG is green. (B) Live-cell images of cells expressing Pik1-D450–mNG with Anp1–mCherry or Sec72–mCherry. (C) Live-cell imaging of cells expressing GFP–P4C_SidC_ with Sec72–mCherry. (D) Live-cell imaging of cells expressing Pik1-D450–mNG with mCherry–Cdc15. Images are representative of two repeats. Scale bars: 5 μm.

To better understand Pik1 function, we aimed to construct an endogenous fluorescently tagged allele. We were not able to recover viable endogenous N- or C-terminally tagged *pik1* alleles*.* Therefore, we designed a construct that inserted mNG within a flexible loop of Pik1 based on the AlphaFold2 predicted structure (Jumper et al., 2021; Varadi et al., 2022) (Fig. 3A). Indeed, we were able to generate a *pik1-D450-mNG* strain, in which mNG was introduced after residue D450 in the endogenous *pik1* locus (Fig. 3A). The insertion is predicted not to disrupt the overall Pik1 fold (Jumper et al., 2021; Mirdita et al., 2022; Varadi et al., 2022) (Fig. 3A), and the *pik1-D450-mNG* strain grew well at a variety of temperatures (Fig. S2B).

To investigate Pik1 localization, we co-imaged Pik1-D450–mNG with either a cis-(Anp1–mCherry) or trans-Golgi (Sec72–mCherry) marker (Vjestica et al., 2008) and found colocalization only with the trans-Golgi marker (Fig. 3B), consistent with *S. cerevisiae* Pik1 localization (Walch-Solimena and Novick, 1999; Sciorra et al., 2005; Strahl et al., 2005). We also found that the Golgi PI4P pool detected by GFP–P4C_SidC_ colocalized with the Sec72–mCherry trans-Golgi marker, consistent with the Pik1 localization (Fig. 3C). We did not detect Pik1-D450–mNG at the cell division site when co-imaged with the CR marker, mCherry–Cdc15 (Fig. 3D). We conclude that Pik1 localizes exclusively to the trans-Golgi and that the previously reported localization to the division site might be explained as an artifact of overexpression (Park et al., 2009).

Additional evidence that Pik1 could be involved in cytokinesis is its reported interaction with the myosin light chains Cam2 and Cdc4 (Desautels et al., 2001; Sammons et al., 2011). When Pik1-D450–mNG was co-imaged with Cam2–mCherry, no colocalization was detected, consistent with Cam2 localizing exclusively to endocytic actin patches in wild-type cells (Fig. S3A) (Sammons et al., 2011). When released from its binding partner, myosin-1, with the *myo1ΔIQ* mutant, Cam2 localizes to non-actin patch puncta (Sammons et al., 2011) that we reasoned might be the Golgi. Thus, we imaged Pik1-D450–mNG and Cam2–mCherry in *myo1ΔIQ* cells; however, we still did not observe colocalization of Cam2 with Pik1 (Fig. S3A). To examine whether a portion of Cdc4 localized to the trans-Golgi, it was co-imaged with Sec72-mCherry; no colocalization was observed at any cell cycle stage (Fig. S3B). We also combined *pik1-11* with a temperature-sensitive allele of *cdc4*, *cdc4-31*, but no genetic interaction was observed (Fig. S3C). These results are consistent with there being a lack of evidence for a role of Pik1 in cytokinesis. Taken together, our data suggest it is unlikely that Pik1 functions with Cdc4 or Cam2 in either cytokinesis or endocytosis.

**Fig. 4.**
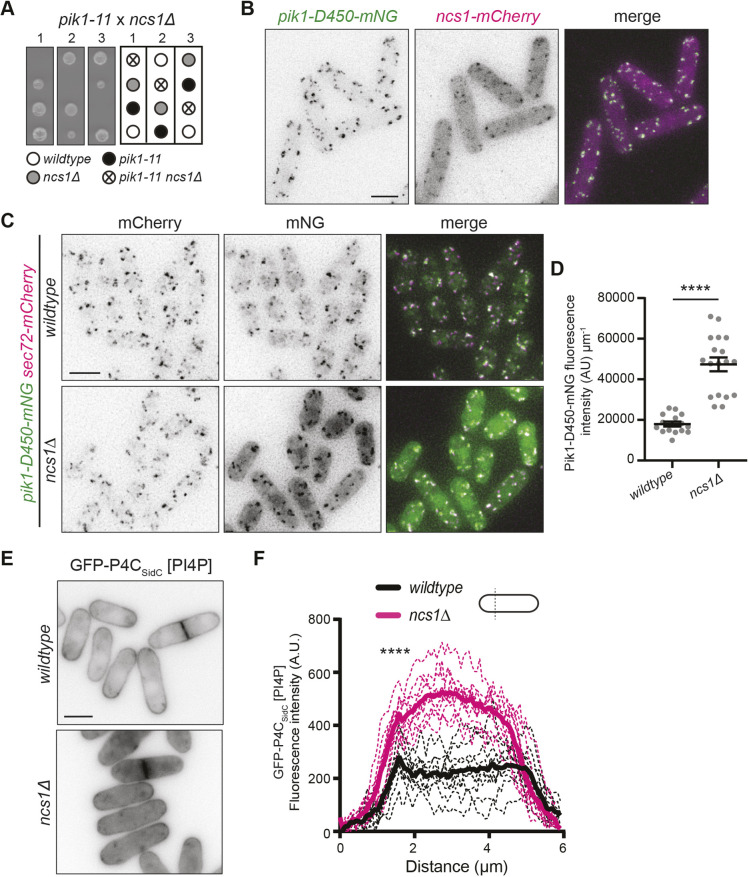
**Ncs1 promotes Pik1 function but is not required for Pik1 localization.** (A) Representative tetrads and schematic of the indicated genetic cross. (B) Live-cell imaging of cells expressing Pik1-D450–mNG and Ncs1–mCherry. (C) Live-cell imaging of cells expressing Pik1-D450–mNG and Sec72–mCherry in wild-type or *ncs1Δ* cells. (D) Quantification of Pik1-D450–mNG whole-cell fluorescence intensity from cells in C. *n*≥15 for each from two biological replicates. ****P≤0.0001 (unpaired two-tailed Student's *t*-test). Error bars represent s.e.m. (E) Live-cell images of GFP–P4C_SidC_ in wild-type or *ncs1Δ* cells. (F) Line scans of fluorescence intensity drawn across the short axis of 10 cells from E for each strain. Solid lines represent the mean and dotted lines are the individual line traces. Data is from two biological replicates. Wild-type versus *ncs1Δ* cells at first peak, 1.58 μm distance, is *P*<0.0001. ****P≤0.0001 (unpaired two-tailed Student's *t*-test). Images in A and B are representative of two repeats. Scale bars: 5 μm.

Ncs1 is an established Pik1-binding partner and regulator (Lim et al., 2011). Therefore, it was not surprising that when we attempted to combine *ncs1Δ* with *pik1-11* we found that they were synthetically lethal (Fig. 4A). Also, as predicted, when Ncs1 was tagged with mCherry, we observed colocalization of Ncs1–mCherry with Pik1-D450–mNG at the trans-Golgi (Fig. 4B). In *S. cerevisiae*, the binding site of the Ncs1 ortholog Frq1 in Pik1 is required for Pik1 Golgi localization implicating Frq1/Ncs1 as necessary for Pik1 Golgi localization (Strahl et al., 2005). Because *ncs1* is not an essential gene (Hamasaki-Katagiri et al., 2004), unlike *S. cerevisiae FRQ1* (Hendricks et al., 1999), it was possible to test this idea in *S. pombe*. We found that Pik1-D450–mNG still localized to the trans-Golgi marked by Sec72–mCherry in *ncs1*Δ cells (Fig. 4C), further evidence of *pik1-D450-mNG* allele function (Fig. S2B). We conclude that, at least in *S. pombe*, Ncs1 is not required for Pik1 Golgi localization. The small GTPase Arf1 is also implicated in promoting *S. cerevisiae* Pik1 Golgi localization (Highland and Fromme, 2021), so perhaps *S. pombe* relies more on this mechanism. Unfortunately, because *S. pombe* Arf1 is essential and conditional alleles of *arf1* are not available, we were unable to test this possibility. Interestingly, there was a high cytoplasmic Pik1 population in *ncs1Δ* cells that was not observed in wild-type cells; indeed, there was >2-fold more Pik1 overall (Fig. 4D). We currently do not have a mechanistic explanation for this observation.

The current model for Frq1/Ncs1 function is that it holds the Pik1 kinase domain in an active conformation (Strahl et al., 2003, 2005, 2007; Lim et al., 2011). To determine whether there was Golgi PI4P in *ncs1*Δ, a proxy for a change in Pik1 activity, we imaged wild-type and *ncs1Δ* cells expressing GFP–P4C_SidC_. We observed the persistence of Golgi PI4P puncta as well as increased cytoplasmic and PM PI4P levels in *ncs1*Δ cells (Fig. 4E,F). Combined with the fact that Pik1 is essential whereas Ncs1 is not (Hamasaki-Katagiri et al., 2004; Park et al., 2009), it seems unlikely that Ncs1 is required for *S. pombe* Pik1 activity and perhaps even acts as a negative regulator. Future studies will be required to dissect the mechanism of Ncs1-dependent regulation of Pik1 in *S. pombe*.

In conclusion, we find that Pik1 is important for Golgi PI4P levels and does not appear to play a role in cytokinesis or impact PM PI(4,5)P_2_ levels under the conditions tested in this paper. Rather, Stt4-generated PI4P is the apparent PI(4,5)P_2_ precursor. Golgi-derived PI4P is thought to be transported to the PM, thus how PI4P molecules synthesized by Stt4 or Pik1 have unique downstream fates is unclear. Lipid enzymes in other organisms have been shown to exist in larger order multi-subunit complexes that encompass multiple biosynthetic steps (Botelho et al., 2008; Jin et al., 2008), and it will be interesting to determine whether a similar mechanism exists in fission yeast that coordinates Stt4 and Its3 activities. Another possibility is that the action of PI-4-phosphatases play an important role in regulating the spatial distribution of PI4P and thus PI(4,5)P_2_ lipid composition. Dissecting these possibilities will be an exciting topic of future studies.

## MATERIALS AND METHODS

### Yeast methods

All *S. pombe* strains used in this study (Table S1) were cultured using standard methods in YE medium (Moreno et al., 1991; Forsburg and Rhind, 2006). Transformation of yeast with linear DNA was accomplished using a lithium acetate method (Keeney and Boeke, 1994; Forsburg and Rhind, 2006). Strain construction was accomplished through tetrad analysis using standard methods.

Tagged *ncs1* strains were generated by inserting sequences encoding mCherry and natMX6 from a pFA6 cassette the 3′ end of the ORF at the endogenous locus as previously described (Wach et al., 1994; Bähler et al., 1998). Nourseothiricin (clonNAT, 100 mg/ml, GoldBio; cat. no. N-500-100) was used for selection of natMX6-containing cells on YE plates. All fusion proteins examined in this study were expressed from their native promoters at their chromosomal loci.

*pik1-D450-mNG* was constructed by cloning synthesized gene blocks (Integrated DNA Technologies) into the PstI site of pIRT2 using Gibson assembly. 300 bp 3′ and 5′ flanks were included as well as the *kanMX6* gene and promoter between the *pik1* stop codon and the 3′ flank. This pIRT2 construct was transformed into cells and G418 (Geneticin, 100 mg/ml, Thermo Fisher Scientific; cat. no. 11811031) was used to select the appropriate integrants on YE plates. Colonies were confirmed with PCR and imaging.

### Isolation of temperature-sensitive alleles with error-prone PCR

The *pik1-11* temperature-sensitive allele was constructed as described previously (Tang et al., 2019) with the exception that EX taq polymerase (Takara, cat. no. 4025) and accompanying dNTPs (Takara, cat. no. RR01BM) were used.

### Microscopy

Yeast cells were grown at 25°C in YE medium prior to live-cell imaging unless otherwise grown at 25°C and then shifted to 36°C for 3 h and imaged at both temperatures. Images were acquired with either (1) a Personal DeltaVision microscope system (Leica Microsystems) that includes an Olympus IX71 microscope, 60×1.42 NA PlanApo oil immersion objective, a 60×1.49 TIRF objective, a pco.edge sCMOS camera and softWoRx imaging software, or (2) with a Zeiss Axio Observer inverted epifluorescence microscope with Zeiss 63× oil (1.46 NA) and captured using Zeiss ZEN 3.0 (Blue edition) software and Axiocam 503 monochrome camera (Zeiss). Images in Figs 2, 4E, Fig. S1B and S1D (bottom images) are non-deconvolved maximum intensity projections of four medial z-sections spaced at 0.5 µm. Images in Figs 2E, 3B,D, Fig. S1D (top images) and S3 are non-deconvolved maximum intensity projections of *z*-sections spaced at 0.5 µm. Images in Figs 3C and 4 are deconvolved maximum intensity projections of *z*-sections spaced at 0.5 µm. Quantification of images was performed using Fiji (a version of ImageJ software available at https://fiji.sc) (Schindelin et al., 2012).

The whole-cell intensity measurement in Fig. 4D was corrected for background. In each image used for quantification, background intensity measurements were taken from an area without any cells, which was divided by that area to give the average intensity per pixel of the background. This value was then multiplied by the area of the region of interest (ROI) and subtracted from the raw intensity measurement for that ROI to get the intensity measurement corrected for background. The corrected intensity measurements were divided by the area of the ROI.

For all line scans, a sum projection of four medial slices of non-deconvolved images were used. Intensity measurements were plotted against the distance across the short cell axis on cells that were 10–12 μm in length. The line scans were aligned by the first peak in fluorescence intensity.

### Protein structure prediction

Protein structure prediction of Pik1-D450-mNG was generated with the ColabFold interface to the AlphaFold2 pipeline on the Colab platform (AlphaFold2.ipynb) (Jumper et al., 2021; Mirdita et al., 2022; Varadi et al., 2022).

### Statistical analysis

All statistical analyses were performed in Prism 8 (Graphpad software). No data were excluded from the analysis.

## Supplementary Material

10.1242/joces.261415_sup1 Supplementary information
