# Supplementary material for: Characterization of Pik1 function in fission yeast reveals its conserved role in lipid synthesis and not cytokinesis
Source: J Cell Sci. 2023 Nov 1;136(21):jcs261415. doi: 10.1242/jcs.261415 (PMC10629694; doi:10.1242/jcs.261415)
Supplement: Supplementary information [file joces-136-261415-s1.pdf]

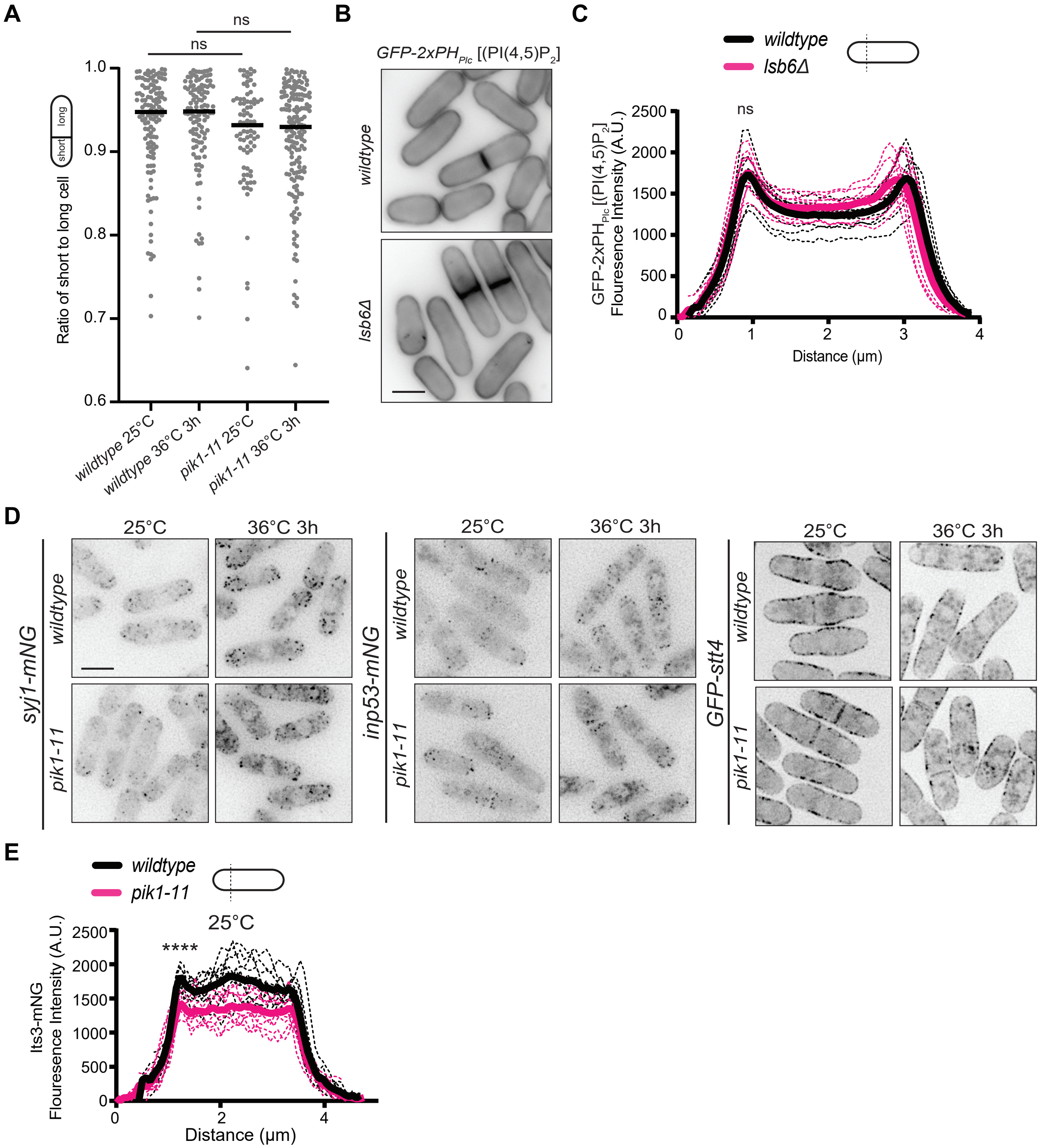

Journal of Cell Science • Supplementary information

Fig. S1. *Isb6Δ* cells do not have reduced PI(4,5)P_2. A) Quantification of septum placement from DIC images of the indicated strains at the indicated temperatures. *wildtype* 25°C vs *pik1-11* 25°C, *P* = 0.558; *wildtype* 36°C vs *pik1-11* 36°C, *P* = 0.106. One-way ANOVA. Data is from two biological replicates and n ≥ 79 for each. B) Live-cell images of GFP-2xPH_{Plc} in wildtype and *Isb6Δ* cells. Cells were grown at 25°C in YE. C, E) Line scans of fluorescence intensity drawn across the short axis of 10 cells for each indicated strain at the indicated temperature. Solid lines represent the mean and dotted lines are the individual line scans. Data is from two biological replicates. For C, *wildtype* vs. *Isb6Δ* at first peak, .93 µm distance, *P* = 0.83. For E, *wildtype* vs. *pik1-11* at first peak, 1.22 µm distance, *P* < 0.0001. **** *P* ≤ .0001, Student's *t*-test. D) Live-cell images of Syj1-mNG, Inp53-mNG or GFP-Stt4 in wildtype and *pik1-11* cells. Cells were grown at 25°C and shifted to 36°C for 3 hours and imaged at both time points. Scale bars, 5 µm.

Journal of Cell Science • Supplementary information

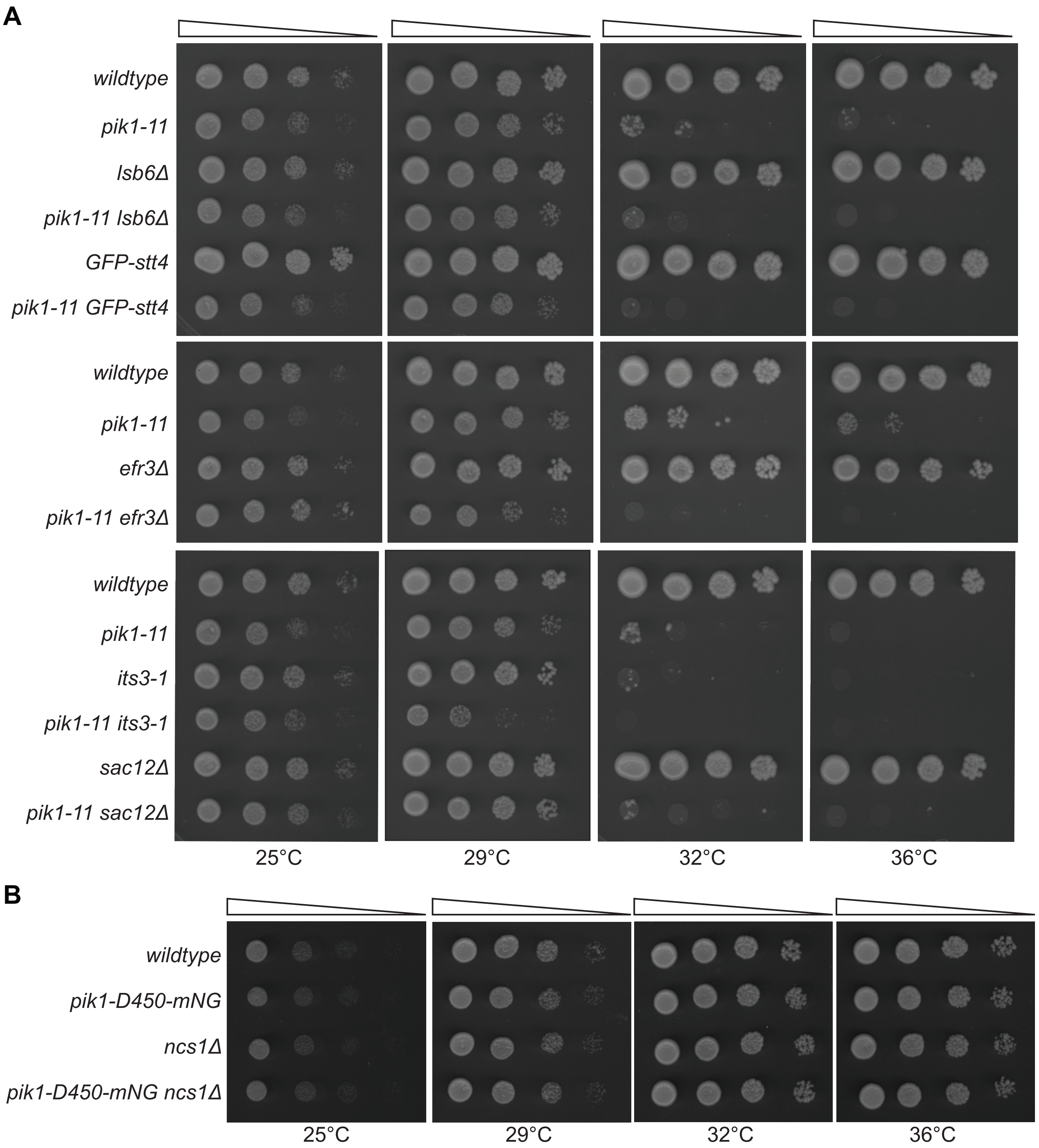

Fig. S2. Genetic analysis of *pik1-11*. A-B) 10-fold serial dilutions of the indicated strains grown at the indicated temperatures on YE agar for 3-4 days.

Journal of Cell Science • Supplementary information

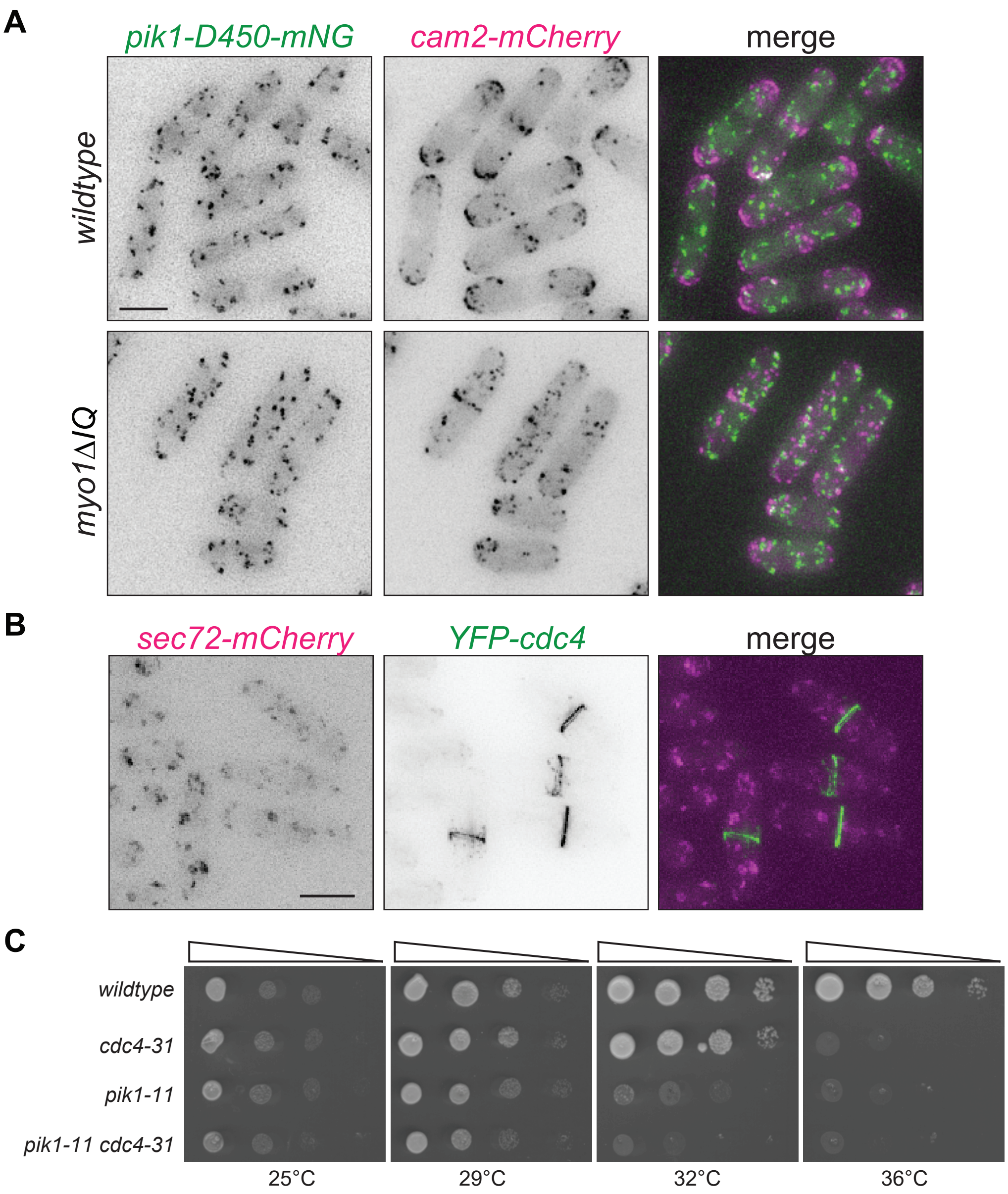

Fig. S3. Cam2 and Cdc4 do not colocalize with Pik1. A-B) Live-cell imaging of the indicated strains. Scale bars, 5 μm. C) 10-fold serial dilutions of the indicated strains grown at the indicated temperatures on YE agar for 3-4 days.

Table S1. *S. pombe* strains used in this study

Strain	Genotype	Source
**Figure 1**		
KGY246	*ade6-M210 ura4-D18 leu1-32 h^{-}*	Lab stock
KGY7427-2	*pik1-11:kanMX6 ade6-M21X ura4-D18 leu1-32 h^{-}*	This study
**Figure 2**		
KGY19217	*GFP-P4C$_{SidC}$:leu1^{+} ade6-M210 leu1-32 ura4-D18 h^{+}*	Lab stock
KGY7593-2	*pik1-11:kanMX6 GFP-P4C$_{SidC}$:leu1^{+} ade6-M210 leu1-32 ura4-D18 h^{+}*	This study
KGY269-2	*GFP-2xPH$_{Plc}$:leu1^{+} ade6-M210 leu1-32 ura4-D18 h^{+}*	Lab stock
KGY7596-2	*pik1-11:kanMX6 GFP-2xPH$_{Plc}$:leu1^{+} ade6-M210 leu1-32 ura4-D18 h^{+}*	This study
KGY243-2	*its3-mNG:hphMX6 ade6-M210 leu1-32 ura4-D18 h^{-}*	Lab stock
KGY8097-2	*its3-mNG:hphMX6 pik1-11:kanMX6 ade6-M210 leu1-32 ura4-D18 h^{-}*	This study
**Figure 3**		
KGY7438-2	*pik1-D450-mNG:kanMX6 anp1-mCherry:ura4^{+} ade6-M210 leu1-32 ura4-D18 h^{+}*	This study
KGY7589-2	*pik1-D450-mNG:kanMX6 sec72-mCherry:ura4^{+} ade6-M210 leu1-32 ura4-D18 h^{-}*	This study
KGY7953-2	*GFP-P4C$_{SidC}$:leu1^{+} sec72-mCherry:ura4^{+} ade6-M210 leu1-32 ura4-D18 h^{+}*	This study
**Figure 4**		
KGY2029-2	*ncs1::kanMX6 ade6-M210 leu1-32 ura4-D18 h^{+}*	Lab stock
KGY7919	*pik1-D450-mNG:kanMX6 ncs1-mCherry:natMX6 ade6-M21X leu1-32 ura4-D18 h^{-}*	This study
KGY7589-2	*pik1-D450-mNG:kanMX6 sec72-mCherry:ura4^{+} ade6-M210 leu1-32 ura4-D18 h^{-}*	This study
KGY7926-2	*pik1-D450-mNG:kanMX6 sec72-mCherry:ura4^{+} ncs1::kanMX6 ade6-M210 leu1-32 ura4-D18 h^{-}*	This study
KGY7461-2	*pik1-D450-mNG:kanMX6 mCherry-cdc15 ade6-M21X leu1-32 ura4-D18 h^{-}*	This study
KGY7955-2	*GFP-P4C$_{SidC}$:leu1^{+} ncs1::kanMX6 ade6-M210 leu1-32 ura4-D18 h^{+}*	This study
**Figure S1**		
KGY3223-2	*syj1-mNG:hphMX6 ade6-M210 leu1-32 ura4-D18 h^{-}*	This study
KGY8066-2	*syj1-mNG:hphMX6 pik1-11:kanMX6 ade6-M210 leu1-32 ura4-D18 h^{+}*	This study
KGY3237-2	*inp53-mNG:hphMX6 ade6-M210 leu1-32 ura4-D18 h^{-}*	This study
KGY8105-2	*inp53-mNG:hphMX6 pik1-11:kanMX6 ade6-M210 leu1-32 ura4-D18 h$^{?}$*	This study
KGY2302-2	*lsb6::kanMX6 GFP-2xPH$_{Plc}$:leu1^{+} ade6-M210 leu1-32 ura4-D18 h^{-}*	This study
**Figure S2**		

KGY18195	*lsb6::kanMX6 ade6-M210 leu1-32 ura4-D18 h*$^{+}$	Lab stock
KGY7751-2	*pik1-11:kanMX6 lsb6::kanMX6 ade6-M210 leu1-32 ura4-D18 h*$^{+}$	This study
KGY18661	*GFP-stt4 ade6-704 leu1-32 h*$^{-}$	Lab stock
KGY7750-2	*GFP-stt4 pik1-11:kanMX6 ade6-704 leu1-32 h*$^{+}$	This study
KGY2025-2	*efr3::ura4*$^{+}$ *ade6-M210 leu1-32 ura4-D18 h*$^{-}$	Lab stock
KGY4622-2	*pik1-11:kanMX6 efr3::ura4*$^{+}$ *ade6-M210 leu1-32 ura4-D18 h*$^{+}$	This study
KGY6369	*its3-1 ade6-M21X leu1-32 ura4-D18 h*$^{-}$	Lab stock
KGY7749-2	*pik1-11:kanMX6 its3-1 ade6-M210 leu1-32 ura4-D18 h*$^{+}$	This study
KGY18307	*sac12::kanMX6 ade6-M210 leu1-32 ura4-D18 h*$^{+}$	Lab stock
KGY7767-2	*pik1-11:kanMX6 sac12::kanMX6 ade6-M21X leu1-32 ura4-D18 h*$^{-}$	This study
KGY7925-2	*pik1-D450-mNG:kanMX6 ncs1::kanMX6 ade6-M210 leu1-32 ura4-D18 h*$^{+}$	This study
**Figure S3**		
KGY7890-2	*cam2-mCherry:natMX6 pik1-D450-mNG:kanMX6 ade6-M21X leu1-32 ura4-D18 h*$^{-}$	This study
KGY7890-2	*myo1ΔIQ1ΔIQ2(unknown) cam2-mCherry:natMX6 pik1-D450-mNG:kanMX6 ade6-M21X leu1-32 ura4-D18 h*$^{-}$	This study
KGY7764-2	*sec72-mCherry:ura4*$^{+}$ *cam2-mNG:hphMX6 ade6-M21X leu1-32 ura4-D18 h*$^{+}$	This study
KGY5091-2	*myo1ΔIQ1ΔIQ2(unknown) sec72-mCherry:ura4*$^{+}$ *cam2-mNeonGreen:hphMX6 ade6-M21X leu1-32 ura4-D18 h*$^{+}$	This study
KGY7949-2	*YFP-cdc4:kanMX6 sec72-mCherry:ura4*$^{+}$ *ade6-M21X leu1-32 ura4-D18 h*$^{-}$	This study
KGY2547-2	*cdc4-31 ade6-M21X leu1-32 ura4-D18 h*$^{-}$	Lab stock
KGY7978-2	*cdc4-31 pik1-11:kanMX6 ade6-M21X leu1-32 ura4-D18 h*$^{-}$	This study

Journal of Cell Science • Supplementary information
